# Repurposing of Anti-Diabetic Agents as a New Opportunity to Alleviate Cognitive Impairment in Neurodegenerative and Neuropsychiatric Disorders

**DOI:** 10.3389/fphar.2021.667874

**Published:** 2021-05-24

**Authors:** Qian Chen, Ting Cao, NaNa Li, Cuirong Zeng, Shuangyang Zhang, Xiangxin Wu, Bikui Zhang, Hualin Cai

**Affiliations:** ^1^ Department of Pharmacy, The Second Xiangya Hospital, Central South University, Changsha, China; ^2^ Institute of Clinical Pharmacy, Central South University, Changsha, China

**Keywords:** cognitive deficits, anti-diabetic agents, brain insulin resistance, alzheimer's disease, schizophrenia, type 2 diabetes mellitus

## Abstract

Cognitive impairment is a shared abnormality between type 2 diabetes mellitus (T2DM) and many neurodegenerative and neuropsychiatric disorders, such as Alzheimer’s disease (AD) and schizophrenia. Emerging evidence suggests that brain insulin resistance plays a significant role in cognitive deficits, which provides the possibility of anti-diabetic agents repositioning to alleviate cognitive deficits. Both preclinical and clinical studies have evaluated the potential cognitive enhancement effects of anti-diabetic agents targeting the insulin pathway. Repurposing of anti-diabetic agents is considered to be promising for cognitive deficits prevention or control in these neurodegenerative and neuropsychiatric disorders. This article reviewed the possible relationship between brain insulin resistance and cognitive deficits. In addition, promising therapeutic interventions, especially current advances in anti-diabetic agents targeting the insulin pathway to alleviate cognitive impairment in AD and schizophrenia were also summarized.

## Introduction

Cognitive deficits can be observed in neurodegenerative and neuropsychiatric disorders, such as AD and schizophrenia with demonstratable brain pathology(Taber et al., 2010). Brain functional and structural abnormalities are related with cognitive deficits. Furthermore, brain changes could be a common biomarker for treatment-related cognitive improvement across neuropsychiatric disorders(Ott et al., 2019). Recent findings demonstrate that brain is a further important site of insulin resistance and brain insulin resistance is associated with cognitive dysfunction(Kullmann et al., 2016). Insulin resistance has been recognized as a mechanism of cognitive dysfunction in T2DM(Kim, 2019). Additionally, in AD, the impaired brain insulin signaling may contribute to cognitive decline via impaired hippocampal neuroplasticity, increased tau protein concentration, neuroinflammation and mitochondrial dysfunction(Biessels and Reagan, 2015). Given the relationship between brain insulin resistance and cognitive impairment, repurposing of already marketed anti-diabetic medications has attracted growing attention as a potential treatment in cognitive decline diseases. Specifically, both preclinical and clinical studies have demonstrated the neuroprotective effects of glucagon-like peptide 1 (GLP-1) receptor agonists, which are used for diabetes and obesity treatment(Mansur et al., 2018).

Accumulating studies have attracted profound scientific and public attention to the greater metabolic comorbidity of T2DM in schizophrenia patients(Mizuki et al., 2020), which is partly owing to overlapping genetic risk factors, environmental susceptibility and antipsychotic drugs-related mechanisms(Suvisaari et al., 2016; Hackinger et al., 2018). Even first-episode drug-naïve patients with schizophrenia exhibited greater insulin resistance compared with normal subjects(Pillinger et al., 2017). Glucose metabolic disturbances may be an inherent part of schizophrenia, and brain insulin resistance is a risk factor for cognitive deficits in this brain disorder(Wijtenburg et al., 2019). Meanwhile, the efficacy of current antipsychotic drugs on cognitive deficits are limited(Hill et al., 2010), making it urgent to develop pro-cognitive strategies. But few investigations have been done to illuminate the precise mechanisms linking insulin resistance and cognitive deficits in schizophrenia. In addition, whether pro-cognitive strategies used in AD via their anti-diabetic effects or other mechanisms in the brain could be generalized to schizophrenia is uncertain. It is of utmost importance to develop and promote effective interventions to ameliorate cognitive deficits in individuals with schizophrenia, further understanding the underlying mechanisms behind cognitive dysfunction and improving the quality of life and clinical outcomes of patients.

This review briefly summarized the mechanisms linking brain insulin resistance and cognitive impairment. In the next section, we provided an overview of preclinical and clinical studies with the aim to explore the potential cognitive enhancement of some anti-diabetic agents in treating AD and schizophrenia.

## Mechanisms connecting brain insulin resistance and cognitive deficits

**FIGURE 1:**
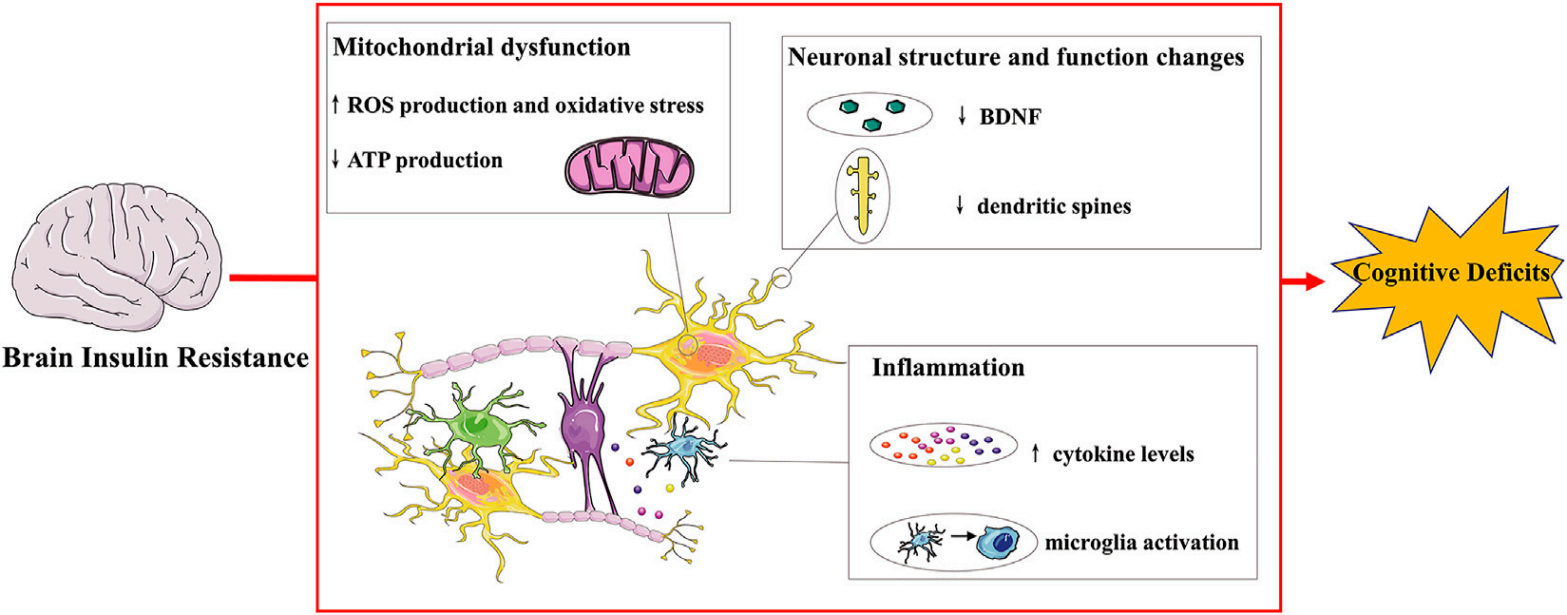
Mechanisms connecting brain insulin resistance and cognitive deficits.Insulin receptors are distributed throughout the brain and their expression and action have also been found in neurons and glial cells. Brain insulin resistance impairs cognitive deficits via multiple ways. For instance, brain insulin resistance affects normal neuronal structure and function, resulting in decreased dendritic spines and BDNF levels and inducing impaired synaptic plasticity and transmission. Brain insulin resistance also exhibits cognitive decline with brain mitochondrial dysfunction with increased oxidative stress, leading to increased ROS and decreased ATP production. Additionally, brain insulin resistance induces raised inflammatory progress: microglia activation and increased cytokine levels, contributing to cognitive deficits. BDNF: brain derived neurotrophic factor; ROS: reactive oxygen species.

Insulin is a peptide hormone secreted by β cells of pancreatic islets, playing a crucial role in metabolism and cognitive function. Insulin resistance refers to a pathological condition of decreased insulin sensitivity of insulin-targeting cells and issues. In periphery, it has been recognized as a main feature of metabolic syndrome and T2DM. The brain is a further important site of insulin resistance, which is an extremely important organ to regulate energy metabolism, body weight, memory and cognition. Insulin receptor expression and action have also been found in neuronal populations and glial cells, and the disruption of insulin signaling in the brain is related to abnormal neuronal function(Kleinridders et al., 2014). In the brain, insulin resistance is associated with impaired neuroplasticity, raised inflammation process and mitochondrial dysfunction, underlining the significant role of brain insulin resistance in cognitive deficits (Figure 1).

### Brain Insulin Resistance and Abnormal Neuronal Structure and Function

Neurons are the structural and functional units of nervous system and brain insulin resistance-induced neuronal injury leads to cognitive deficits. Brain insulin resistance is associated with reduced dendritic spines, decreased level of BDNF and impaired synaptic plasticity(Biessels and Reagan, 2015; Park et al., 2019).

Dendritic spines are small postsynaptic protrusive structures from a dendritic, which are essential for synaptic transmission, and ultimately for learning and memory function(Nakahata and Yasuda, 2018). Hippocampal dendritic spines are also regulated by BDNF and brain insulin resistance decreases hippocampal dendritic spine density and disrupts the production of BDNF(Ding et al., 2017), leading to reduced LTP in the hippocampus and impaired cognitive function(Xiang et al., 2015). The decreased level of BDNF in insulin resistant brain would affect mitochondrial and protein synthesis, influencing synaptogenesis and neuronal health(Tome-Carneiro et al., 2018). BDNF is secreted in response to stimulations and is dependent on calcium influx through voltage gated calcium channels or N-methyl-d-aspartate (NMDA) receptors(Brigadski and Leßmann, 2020). Insulin resistance in the brain disrupted the NMDA receptor phosphorylation and final the production of BDNF(Ding et al., 2017). Besides, neuronal insulin resistance induced the down-regulation of cyclic element binding protein (CREB) and the expression of BDNF(Mi et al., 2017). And the decreased insulin resistance could stimulate the increased release of BDNF into the serum(Śmieszek et al., 2017).

Synaptic plasticity refers to numeral, structural and functional modifications of synapses under various stimulations, leading to the corresponding changes of the transmission efficacy(Citri and Malenka, 2008). In addition to resulting in decreased dendritic spines and reduced hippocampal BDNF levels, insulin resistance can cause deficits in brain synaptic plasticity, which together leading to impairment of cognitive function. Rats with specific downregulation of hippocampal insulin receptors by using a lentiviral vector exhibited impaired spatial learning and memory function through alterations in the expression and phosphorylation of glutamate receptor subunits(Grillo et al., 2015). Disruption of IRS-2 in the mice hippocampus impaired NMDA receptor-dependent LTP of synaptic transmission(Martin et al., 2012). Additionally, insulin resistance impaired synaptic plasticity and memory function through the hyper-palmitoylation of α-amino-3-hydroxy-5methyl-4-isoxazole propionic acid (AMPA) glutamate receptor subunit GluA1 in the hippocampus(Spinelli et al., 2017). An impaired insulin signaling in the brain also changed integrin-linked kinase glycogen synthase kinase (GSK) 3β signaling and reduced the trafficking and function of postsynaptic glutamate receptors, thereby impairing synaptic plasticity and contributing to cognitive decline(Shonesy et al., 2012).

### Brain Insulin Resistance and Inflammation

Inflammation is the defensive response to stimuli occurring in the body and brain. In neuropathological disorders, raised inflammatory process (microglia activation and elevated cytokine levels) impairs cognitive performance through disrupting neurobiological mechanism: synaptic plasticity, neurogenesis, neurotrophic factors, the HPA axis and the kynurenine pathway(Fourrier et al., 2019). For instance, pro-inflammatory cytokines induce the activation of hippocampal indoleamine 2,3-dioxygenase, a tryptophan-catabolizing enzyme in the kynurenine pathway, which participates in learning and memory function(André et al., 2008; Too et al., 2016). The raised inflammatory process may promote the production of kynurenine, the NMDA antagonist, resulting in glutamatergic transmission dysregulation and eventually leading to cognitive impairment in schizophrenia(Müller, 2008). Additionally, immune-mediated imbalance of tryptophan catabolism via the kynurenine pathway is also associated with neuroinflammatory neurological disorders including AD(Maddison and Giorgini, 2015). The activation of the kynurenine pathway induced by inflammatory cytokines may generate neurotoxic metabolites including quinolinic acid and kynurenic acid, which are likely to play a role in the pathogenesis of AD(Gong et al., 2011).

Brain insulin resistance is associated with inflammation. High fat diet (HFD) rodents showed cognitive impairment with elevated Interleukin-1β (IL-1β)(Almeida-Suhett et al., 2017) and tumor necrosis factor α (TNFα) in the hippocampus(Boitard et al., 2014). Meanwhile, proinflammatory cytokines such as TNFα promoted the development of insulin resistance via interference with intracellular pathways(Shoelson et al., 2006). TNFα induced the activation of NF-κβ signaling pathway and caspase 3 was associated with diabetic-induced cognitive deficits and insulin combination with tocotrienol exhibited promising cognitive enhancement in the diabetic rats(Kuhad et al., 2009). The dysregulated insulin signaling in the hippocampus activated microglia and astrocyte, decreased BDNF levels, and inhibited neurogenesis, leading to cognitive deficits(Liu et al., 2018).

### Brain Insulin Resistance and Mitochondrial Dysfunction

Mitochondria are essential for neuronal activity and plasticity, and mitochondrial dysfunction leads to cortical under-connectivity: reduced dendrite, axon and synapse growth(Fernandez et al., 2019). Meanwhile, abnormalities in mitochondrial structure and function have been observed in psychiatric disease(Clay et al., 2011), and cell-free mitochondrial DNA fragment could be used as a biomarker for cognitive deficits in schizophrenia(Suárez-Méndez et al., 2020).

Insulin resistance is related with mitochondrial dysfunction and mitochondria-dependent high level of free radicals also induce insulin resistance(Yaribeygi et al., 2019). Rodents with brain-specific knockout of the insulin receptors exhibited brain mitochondrial dysfunction and dopamine dysfunction leading to behavioral disorder(Kleinridders et al., 2015). HFD-induced brain insulin resistance reduced mitochondrial ATP production rate and oxidative enzyme activities, increased ROS emission and oxidative stress(Ruegsegger et al., 2019). The decreased insulin signaling in the hippocampus resulted in cognitive decline accompanied with decreased mitochondrial oxidative phosphorylation complex proteins(Petrov et al., 2015). Additionally, the activation of insulin receptors and subsequent activation of the α submit of AMP-activated protein kinase (AMPK) improved brain mitochondrial biogenesis(Barhwal et al., 2015).

Although the basic pathologies of AD and schizophrenia are diverse, they still have some symptomatic similarities in addition to cognitive deficits(White and Cummings, 1996), suggesting a complex relationship between the two diseases(Youn et al., 2011). Moreover, new findings support a genetic liability between schizophrenia and psychosis in AD(Creese et al., 2019). For instance, various psychiatric symptoms such as positive and negative symptoms are highly prevalent in both AD and schizophrenia(White and Cummings, 1996). Additionally, some brain morphological data indicate that the neurodegenerative features like progressive brain tissue loss can also occur in patients with schizophrenia(Rund, 2009). The clinical and pathophysiologic analogies of AD and schizophrenia suggest that there are maybe some shared pathological underpinnings between these disorders, which may provide additional insights into the mechanisms underlying both disorders(White and Cummings, 1996). Brain insulin resistance may be one of the referred underpinnings. Compared with the general population, patients with AD are more vulnerable to T2DM(Janson et al., 2004). The Mayo Clinic Alzheimer Disease Patient Registry revealed that 81% of AD patients had either T2DM or an impaired fasting glucose level(Janson et al., 2004). Meanwhile, there is a 2- to 5-fold higher risk of T2DM in people with schizophrenia than in the general population(Ward and Druss, 2015). Although the original causes for cognitive impairment in AD and schizophrenia are somewhat different, similar disturbances in glucose metabolism and insulin signaling will undoubtedly have further negative impact on the cognitive function of both disorders. A study demonstrated that insulin sensitivity as indexed by HOMA value was negatively coupled with verbal fluency performance, brain size and temporal lobe gray matter volume in regions known to be involved in speech production in cognitively healthy, nondiabetic elderly men and women(Benedict et al., 2012). Furthermore, higher level of insulin resistance was an independent predictor of poor verbal fluency performance(Ekblad et al., 2017). As mentioned above, it is plausible that brain insulin resistance contributes to cognitive deficits in schizophrenia. A systematic review supported that metabolic syndrome including insulin resistance were associated with cognitive impairment in schizophrenia(Bora et al., 2016). Meanwhile, higher levels of glucose and insulin resistance were found in first-episode drug-naïve patients with schizophrenia who showed cognitive deficits and disruption of white matter structure(Zhang et al., 2019). The level of insulin resistance was elevated and correlated with the severity of cognitive impairment in first-episode drug-naïve patients with schizophrenia(Tao et al., 2020). However, lack of studies directly detects whether individuals with schizophrenia show brain insulin resistance. Recently, a study demonstrated the insulin signaling abnormalities in neuronal cells in first-episode drug-naïve patients with schizophrenia(Kapogiannis et al., 2019). Another study using magnetic resonance spectroscopy (MRS) to detect brain glucose metabolism found a relationship between lower brain glucose utilization and decreased memory measures in schizophrenia patients compared to controls(Wijtenburg et al., 2019). They also detected brain insulin resistance by blood Extracellular Vesicle (EV) biomarkers to verify the association between neuronal insulin resistance and brain glucose metabolism(Wijtenburg et al., 2019). It suggests that brain insulin resistance and glucose metabolism alteration play a part in the pathophysiological of cognitive dysfunction in schizophrenia. Central insulin action is relevant to cognition and cognitive dysfunction in schizophrenia could be linked to central insulin defects(Agarwal et al., 2020). Furthermore, drugs that can act as insulin sensitizers and/or bypass insulin resistance in the brain offer a unique opportunity to address cognitive deficits and improve lives of the patients with schizophrenia(Agarwal et al., 2019).

## Repurposing of anti-diabetic agents as a potential treatment targeting cognitive function in AD and schizophrenia

**FIGURE 2:**
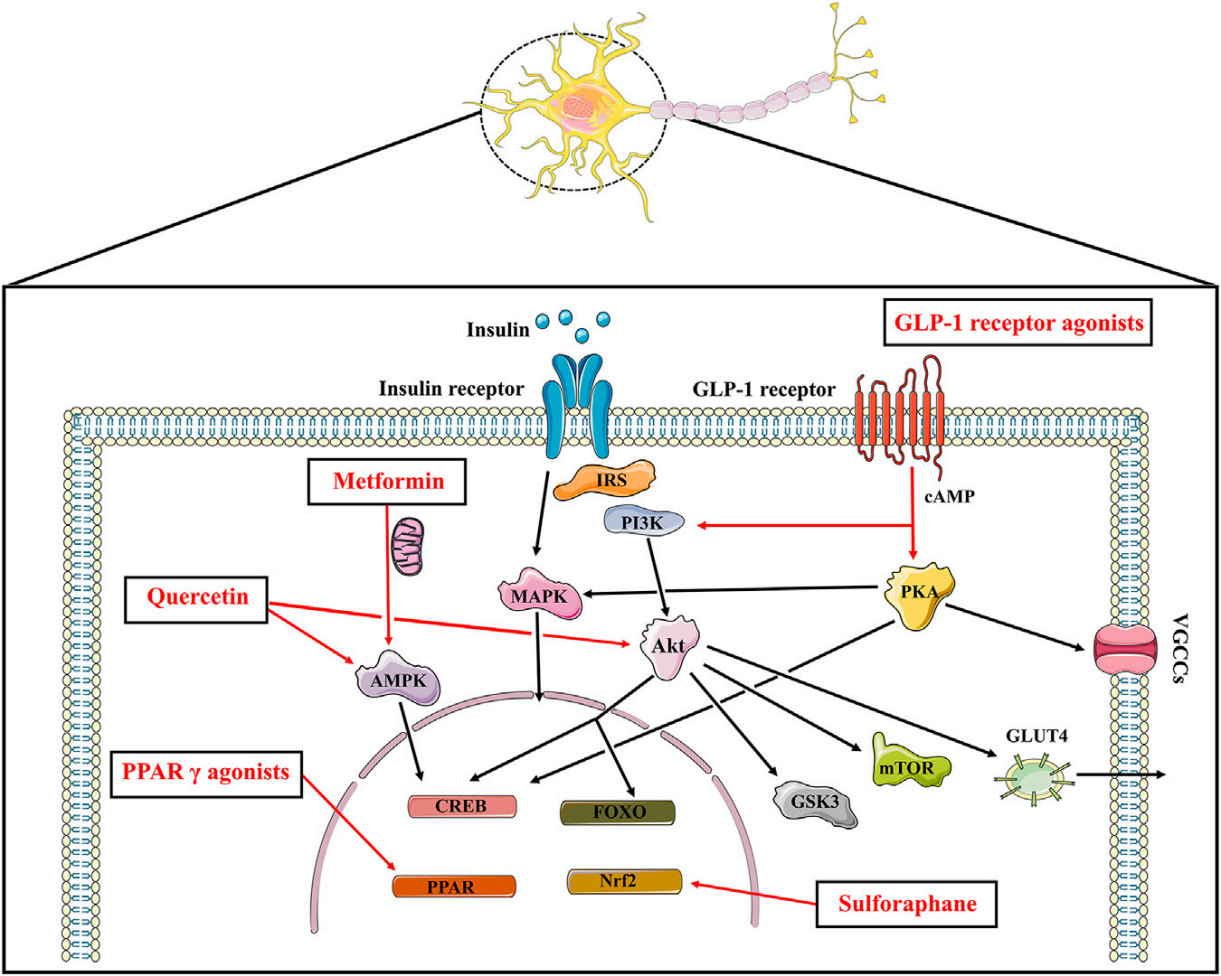
The insulin signaling and the potential pro-cognitive effects of anti-diabetic agents. Insulin binds to insulin receptor to play its part, which promotes PI3K and Akt, the major downstream nodes of insulin signaling. The downstream targets of Akt such as mTOR, GSK3, CREB as well as FOXO play a role in cognitive function. Insulin stimulated phosphorylation of Akt also affects the translocation of the insulin-sensitive glucose transporter GLUT4 to the plasma membrane. In addition to the PI3K/Akt cascade above, insulin activates the MAPK pathways. Anti-diabetic agents boosting insulin signaling exhibit promising pro-cognitive effects. Metformin inhibits mitochondrial complex Ⅰ, thereby increasing AMP/ATP ratio and activating AMPK to protect neurons from oxidative stress. Metformin also upregulates the expression of BDNF via the activation of AMPK and CREB. GLP-1 agonists increase cAMP levels followed by activating PI3K/Akt signaling and PKA signaling pathways. Pioglitazone and rosiglitazone activate PPAR γ, resulting in gene transcription and neuroprotective effects. Sulforaphane exhibits promising neuroprotective effects which is known for an activator of Nrf2-antioxidant response element pathway. Quercetin also enhances cognitive function through its AMPK activity and modulating Akt signaling. IRS: insulin receptor substrate; PI3K: phosphoinositide 3-kinase; Akt: protein kinase B; CREB: cAMP response element binding protein; FOXO: forkhead box transcription factors of the class O; mTOR: mammalian target of rapamycin kinase; GSK3: glycogen synthase kinase 3; GLUT: glucose transporter; MAPK: mitogen-activated protein kinase; AMPK: AMP-activated protein kinase; GLP: glucagon-like peptide; PKA: protein kinase A; VGCCs: voltage gated calcium channels; PPAR: peroxisome proliferator-activated receptors.

**FIGURE 3:**
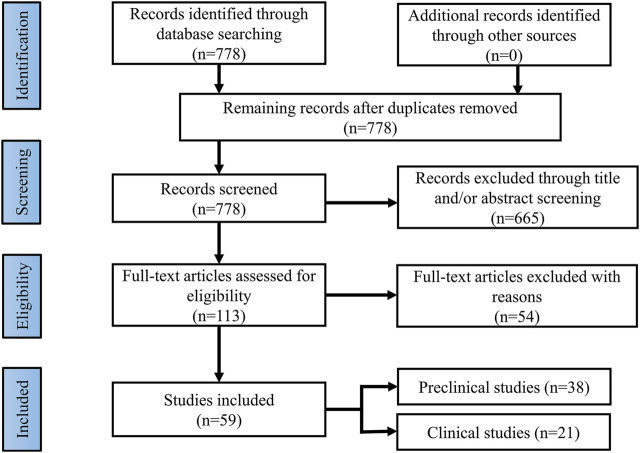
Flowchart of study selection

Current findings suggest that brain insulin resistance is associated with the development of cognitive deficits and anti-diabetic agents boosting insulin action in the brain present promising cognitive enhancement in T2DM and neurodegenerative diseases. Based on this opinion, targeting brain insulin resistance may have therapeutic potential to alleviate cognitive deficits in individuals with schizophrenia (Figure 2). Therefore, we summarized several anti-diabetic drugs targeting the insulin pathway to improve cognitive function which could be pro-cognitive therapeutic interventions in AD and schizophrenia. Literature research (Figure 3: Flowchart of study selection) was conducted on PubMed (last: 8 April 2021) with the combinations of the key words: antidiabetic agents, cognitive impairment, Alzheimer disease, schizophrenia, neurodegenerative disorders, and neuropsychiatry. Inclusion criteria were: (1) animal models or patients with cognitive deficits; (2) under the treatment or supplement treatment of antidiabetic agents; (3) outcomes involved in cognitive function. Exclusion criteria were: (1) a review or a letter; (2) non-English language; (3) streptozocin-induced diabetic animal models. Totally, 38 preclinical and 21 clinical studies were selected for this review (see Table 1 and Table 2 for details).

**TABLE 1:** Preclinical studies: targeting insulin pathway to improve cognitive deficits

Refs	Drug	In vitro/vivo models, dose used, and intervention period	Key Findings i: Biochemical test; ii: Gene and protein expression analysis; iii: Immunohistochemistry and image analysis; iv: Electrophysiology analysis; v: Behavioural assessment;	Brief Conclusions
Farzampour et al. (2016)	Intranasal insulin	Aβ-induced rat model of AD received normal saline or insulin (0.1, 0.2, and 0.3 IU) for 14 consecutive days.	v: (0.2 and 0.3 IU) ↑ working and reference memory: MWM;	Intranasal insulin treatment improved memory and learning in a rat amyloid-beta model of Alzheimer’s disease.
Chen et al. (2014)	Intranasal insulin	9-month-old 3xTg-AD mice received intranasal insulin 1.75 U/17.5 μl or vehicle for 7 days.	ii: restored the insulin signaling in the brain: IR, IGF-1R, IRS-1, PI3K, PDK1 and AKT; ↑ the level of synaptic proteins: synapsin1, PSD95, synaptophysin; ↓ Aβ40 level and microglia activation;	Daily intranasal insulin into 3xTg-AD mice for 7 days restored insulin signaling, increased synaptic proteins, and reduced Aβ40 level and microglia activation in the brain.
Mao et al. (2016)	Intranasal insulin	APP/PS1 mice received intranasal insulin 1 U/day or vehicle for 6 weeks.	ii: improved aberrant insulin signaling in the brain : IRβ, IGF1R, IRS1, PDK1, AKT; ↓ the activation of JNK;	Intranasal insulin treatment for 6 weeks could decrease anxiety-related behaviors, ameliorate cognitive deficits, enhance the impaired brain insulin signaling, alleviate of Aβ pathology and promote neurogenesis.
			iii: ↓ the area of Aβ plaque in the brain; ↑ the number of doublecortin-immunoreactive cells;	
			v: ↓ anxiety and ↑ spatial learning and memory function: OFT, MWM;	
Gupta et al. (2011)	Metformin	Neuro-2a cells exposed to hyperinsulinemic condition before treatment with metformin (0-3.2 mM) for 24 h or 48 h.	i: ↑ glucose uptake: ↑ 2-DOG uptake (maximum 1.6 mM);	Metformin ameliorated neuronal insulin resistance and AD-neuropathological changes; activated AMPK.
			ii:↑ phosphorylation of IRβ, IRS1, PI3K, Akt (1.6 mM); ↓ tau phosphorylation, tau kinase, GSK3β, ERK 1/2, FAK phosphorylation, amyloid-β;↑ insulin-stimulated PKCζ phosphorylation; ↓ Ache activity; ↓ NF-κB translocation to the nucleus; ↑ AMPK phosphorylation (1.6 mM);	
Keshavarzi et al. (2019)	Metformin	Male Wistar rats received normal saline or methamphetamine (10 mg/kg), or methamphetamine (10 mg/kg) plus metformin (50, 75, 100, 150 mg/kg).	In methamphetamine treated rats	Metformin protected the brain against from methamphetamine-induced neurodegeneration through mediating CREB/BDNF or Akt/GSK3 signaling pathway.
			i: ↓ the activity of antioxidant enzymes: SOD, GPx and GR (75, 100, 150 mg/kg);	
			ii: ↑ CREB, BDNF expression/level (75, 100, 150 mg/kg), ↑ Akt expression and inhibited GSK3 expression/level (100, 150 mg/kg), ↓ the level of inflammatory biomarkers: TNF-α and IL-1β (100, 150 mg/kg) in the hippocampus;	
			v: protected rats from anxiety, depression, cognition impairment and motor activity disturbances: OFT, FST, EPM, TST, OFT, MWM;	
Ou et al. (2018)	Metformin	APP/PS1 female mice received 200 mg/kg metformin i.p. for 14 days and wild type littermates were injected with saline.	In APP/PS1 mice:	Metformin could alleviate amyloidogenesis and inflammatory responses, and improve spatial memory, neuroprotection, neurogenesis of the hippocampus in APP/PS1 mice.
			ii: ↓ brain Aβ deposition and Aβ levels; ↓ inflammatory cytokine levels (IL-1β and TNF-α); ↑ the levels of AMPK, ↓ p-mTOR, p-S6K, p-P65NFκB and BACE 1;	
			iii: ↓ neuronal cell death, ↑ neurogenesis, ↓ inflammatory reaction (astrocytic and microglia reactivity);	
			v: rescued spatial memory deficits: MWM;	
Lu et al. (2020)	Metformin	7-month-old male APP/PS1 mice (C57BL/6) received (200 mg/kg p.o.) metformin for 8 weeks.	In APP/PS1 mice	Metformin could relieve learning and memory dysfunction and improve brain function in APP/PS1 mice.
			i: ↓ brain oxidative stress and inflammation: MDA and SOD; IL-1β and IL-6;	
			ii: ↓ brain Aβ accumulation; ↑insulin-degrading enzyme, neprilysin, and p-AMPK expression;	
			iii: ↑ brain function: ↑18F-Fluordeoxyglucose uptake (microPET-CT);	
			v: ameliorated learning and memory dysfunction: MWM and Y-maze tests;	
Farr et al. (2019)	Metformin	SAMP8 mouse received daily injections of metformin at 20 mg/kg/sc or 200 mg/kg/sc for 8 weeks.	In SAMP8 mouse:	Metformin at 20 and 200 mg/kg improved memory in 12-month-old SAMP8 mice.
			i: ↑ PKC (20 mg/kg);	
			ii: ↑ pGSK-3βser (200 mg/kg); ↓ Aβ (20 mg/kg); ↓ pTau and APPc99 (20 and 200 mg/kg);	
			v: ↑ learning and memory: T-maze and NOR;	
Wang et al. (2020)	Metformin	APP/PS1 mouse were injected intraperitoneally with metformin (200 mg/kg/day) or saline for 10 days.	ii: prevented Cdk5 hyperactivation (↓ phosphorylation level of histone H1); inhibited cleavage of p35 into p25;	Metformin could inhibit Cdk5 activity to restore spine density, surface GluA1 trafficking, LTP expression and spatial memory to those of normal level in the APP/PS1 mice.
			iii: corrected the dendritic spine density to control level; rescued AMPA submit GluA1 expression;	
			iv: reversed the decreased f EPSP input-output; rescued LTP defects;	
			v: rescued spatial memory deficits: MWM;	
Wang et al. (2019)	Metformin	Male SD rats received normal saline or MK-801 (0.1 mg/kg, twice-daily, i.p., 2 weeks) and then the MK-801 group randomly received vehicle, metformin (300 mg/kg) or olanzapine (4 mg/kg), i.p., for 4 weeks.	ii: (metformin) ↓ the MK-801 induced higher phosphorylation of Akt and GSK3β;	Metformin reversed MK-801 induced schizophrenia-like symptoms (PPI deficits, hyperactivity, anxiety-like symptoms, recognition and spatial memory impairment).
			v: (metformin or olanzapine) alleviated MK-801 induced PPI deficits, hyperactivity (OFT), cognition memory and spatial learning deficits (MWM); (metformin) alleviated MK-801 induced anxiety like behaviors (elevated plus maze test);	
Chen et al. (2012)	GLP-1 or exendin-4	PC12 cells were treated with high glucose or hydrogen peroxide for 96 h before treatment with GLP-1 or exendin-4 (50, 100, 200, and 1,000 nM) for 96 h.	100 nM GLP-1 or exendin-4	GLP-1 and exendin-4 inhibited high glucose-induced apoptosis and oxidative stress in neurons.
			ii: ↓ the elevated Bax/Bcl-2 ratio in high glucose-induced neurotoxicity;	
			iii: ↑ cell viability in H_2_O_2_ triggered cytotoxicity;	
Zhou et al. (2019)	Dulaglutide	STZ-induced AD-like mice or control mice received vehicle, dulaglutide (0.6 mg/kg/week i.p.), or dulaglutide and exendin (9-39) (0.67 mg/kg/week i.p.) for 4 weeks.	i: no effects on blood glucose; dulaglutide ↓ body weight;	Dulaglutide ameliorated STZ-induced AD-like impairment of learning and memory ability by modulating hyperphosphorylation of tau and neurofilaments through PI3K/AKT/GSK3βsignaling pathway.
			ii: dulaglutide ↓ the phosphorylation levels of tau and neurofilaments; dulaglutide ↑ the expression of GLP-1 and GLP-1R expression; dulaglutide ↑ PI3K/AKT/GSK3β pathway in the STZ mice brain;	
			v: dulaglutide ↑ learning and memory impairment of AD-like mice: MWM;	
Batista et al. (2018)	Liraglutide	Male Swiss mice received daily i.p. injections of liraglutide (25 nmol/kg) or vehicle (PBS) for 7 days before received the injection of Aβ oligomers (Aβ O) (10 pmol) or vehicle into the lateral ventricle; Four Aβ O-injected non-human primates, two Aβ O-injected non-human primates that received liraglutide treatment and three controls.	In Aβ O-injected mice:	Liraglutide reversed cognitive impairment and IR loss caused by AβOs in mice, and it also exerted partial neuroprotective actions in non-human primates.
			ii: ↑ PKA activity; preserved hippocampal level of IRα mRNA;	
			v: prevented memory impairment: NOR, the object location memory test;	
			In Aβ O-injected non-human primates:	
			ii: ↑ IRα and IR β in the frontal cortex; ↑IRα in the hippocampus; attenuated Aβ O-induced AD-like tau phosphorylation;	
			iii: ↑ synaptosin, PSD 95 and density of synapse in the hippocampus, frontal cortex and amygdala;	
McClean et al. (2011)	Liraglutide	7-month-old APP/PS1 mice and wild type controls received saline or liraglutide (25 nmol/kg body weight i.p. once daily) for 8 weeks.	In APP/PS1 mice:	Liraglutide prevented neurodegenerative development in a mouse model of AD.
			iii: ↓ Aβ formation; ↓ microglia activation; ↑ synaptophysin levels; ↓ Aβ oligomer and total brain APP levels;	
			iv: ↑ induction and maintenance of LTP;	
			v: prevented memory impairment: object recognition; MWM;	
Xiong et al. (2013)	Liraglutide	STZ-induced AD-like memory and learning impairment mice received liraglutide (300 ug/kg s.c.) for 30 days.	i: no effects on blood sugar levels;	Liraglutide exhibited neuroprotection effects on STZ-induced AD-like memory and learning impairment mice by modulating the hyperphosphorylation of tau and neurofilament proteins and insulin signaling.
			iii: ↓ hyperphosphorylation of neurofilaments in the brain; ↓ hyperphosphorylated tau in the brain; ↑ microtubule binding tau impaired by STZ; ameliorated ERK and JNK signaling;	
			v: ↑ learning and memory impairment: MWM;	
Hansen et al. (2015)	Liraglutide	6-month-old senescence-accelerated mouse prone 8 (SAMP8) mice received liraglutide (100 or 500 ug/kg/day, s.c.) or vehicle once daily for 4 months.	In SAMP8 mice:	Liraglutide delayed or partially halted the progressive decline in memory function associated with hippocampal neuronal loss in SAMP8 mice.
			i: no effects on body wight, food intake;	
			iii: preserved hippocampal CA1 pyramidal neurons;	
			v: ↑ memory retention: active-avoidance T-maze task; no effects on NOR tests;	
McClean et al. (2015)	Liraglutide	2-months-old APP/PS1 mice received liraglutide (25 nm/kg bw i.p.) or vehicle for 8 months.	In APP/PS1 mice:	Liraglutide reduced AD progressive neurodegeneration in the APP/PS1 mouse model.
			i: no effects on body wight and plasma glucose;	
			iii: ↓ plaque load and inflammatory response (microglia activation); ↑ synaptophysin levels;	
			iv: ↑ LTP;	
			v: ↑ cognition: MWM; maintained recognition memory: NOR; no effects on the open field tests;	
Duarte et al. (2020)	Liraglutide	10-month-old 3xTg-AD female mice received liraglutide (0.2 mg/kg, once/day) for 28 days.	In 3xTg-AD mice:	Liraglutide partially attenuated brain estradiol and GLP-1 and activated PKA levels, oxidative/nitrosative stress and inflammation in these AD female mice.
			i: normalized plasma inflammatory markers; promoted brain glucose metabolism;	
			ii: ↓ brain Aβ_1-42_, Aβ_1-40_ and p-tau; partly normalized brain levels of estradiol and GLP-1-related signaling; rescued brain oxidative/nitrosative stress markers;	
			v: limited signs of cognitive changes: MWM;	
Robinson et al. (2019)	Insulin combination with exenatide	Tg2576 mice received 8-month intranasal administration of insulin and exenatide (0.43×10^−3^ IU + 0.075 μg exenatide + 5 μg BSA per mouse once daily).	The combination of insulin and exenatide:	Combination of insulin with exenatide was associated with better memory and normal expression of insulin receptor pathway genes in a mouse model of AD.
			ii: normalized expression of insulin receptor pathway genes; no effects on Aβ levels;	
			v: ↑ spatial learning but did not reach significance: MWM;	
Bomba et al. (2018)	Exenatide	10 months age adult mice received exenatide (500 mg/kg, bw, i.p.) or vehicle 5 days per week for 2 months.	i: no effects on body wight, fasting glycemia;	Exenatide improved age-dependent cognitive decline through promoting the BDNF-TrkB neurotrophic axis and inhibiting apoptosis by activating decreasing p75NTR-mediated signaling.
			ii: ↑ expression of BDNF and phosphorylation of BDNF, TrkB, ERK5 and PSD95 in the hippocampus; ↓ the expression of pro BDNF, p75NTR, and phosphorylated ERK1,2 (pERK1,2) and JNK (p JNK);	
			iii: ↑ dendritic spine density in hippocampal neurons;	
			v: ↑ long-term memory performance: MWM;	
An et al. (2019)	Exenatide	5-month-old male 5xFAD transgenic AD mice received exenatide s.c. for 16 weeks (100 ug/kg twice per day).	In 5xFAD mice:	Exenatide treatment could improve cognitive impairment, reduce Aβ_1-42_ deposition, alleviate synaptic degradation, improve mitochondrial morphology, relieve oxidative stress, correct the crisis of mitochondrial energy production and normalize mitochondrial morphology in 5xFAD transgenic AD mice.
			ii: ↓ MDA level and ↑ SOD activity; ↑ ATP level and respiratory chain complex Ⅰactivity;	
			iii: ↓ Aβ deposition in the hippocampal CA1 region; alleviated synaptic degradation in the hippocampus; ↑ mitochondrial morphology in the hippocampus; normalized mitochondrial dynamics;	
			v: ↑ learning ability and spatial memory ability: MWM;	
Bomba et al. (2019)	Exenatide	6-month-old 3xTg-AD mice received control or HFD before treatment with exenatide (500 ug/kg body weight, i.p., 5 days per week) or vehicle for 3 months.	In 3xTg-AD mice HFD mice:	Exenatide reverted the adverse changes of BDNF signaling and neuroinflammation status of 3xTg-AD mice undergoing HFD without affecting systemic metabolism or promoting changes in cognitive performances.
			i: no effects on body wight, glucose metabolism;	
			ii: no effects on the levels of Aβ and tau; ↑ the level of BDNF, pERK5, pCREB, pSyn, PSD95 and pTrkB; reverted the HFD-induced activation of proBDNF/p75NTR signaling;	
			v: no effects on learning and memory function: MWM;	
Chen et al. (2019b)	Sitagliptin and saxagliptin	3xTg mice received sitagliptin (3.5 mg/kg s.c.) and saxagliptin (2.6 mg/kg s.c.) for 56 days.	In 3xTg-AD mice HFD mice:	DPP-4 inhibitors improved the impaired spatial learning and memory, decreased tau and NFs aggregation, increased Aβ degradation and reversed AD-like neurodegeneration through partial improvement of GLP-1 signaling pathway including PI3K-Akt and MAPK.
			ii: ↑ the level of GLP-1 and GLP-1R in the brain; ↑ the synapse protein level and activated CREB; modulated the phosphorylation and O-Glycosylation of tau and neurofilaments protein; ↑ GLP-1 signaling;	
			v: ↑ spatial learning and memory ability: MWM;	
Kosaraju et al. (2013a)	Saxagliptin	Streptozotocin-induced rat model of AD received saxagliptin (0.25, 0.5 and 1 mg/kg p.o.) for 60 days	i: ↑ the level of GLP-1;	Saxagliptin attenuated Aβ burden, tau phosphorylation, inflammation and reversed behavioural deficits in streptozotocin-induced rat model of AD.
			ii: ↓ Aβ_1-42_ in the hippocampus; ↓ total tau and p-tau in the hippocampus; ↓ the level of TNF-α and IL-1β in the hippocampus;	
			iii: ↑ cresyl violet-positive neurons in the hippocampus;	
			v: ↑ learning and memory: radial arm maze task; hole-board task;	
Kosaraju et al. (2013b)	Vildagliptin	Streptozotocin-induced rat model of AD received vildagliptin (2.5, 5 and 10 mg/kg p.o.) for 30 days.	ii: ↑ the level of GLP-1; ↓ Aβ_42_ in the brain; ↓ p-tau in the brain; ↓ the level of TNF-α and IL-1β in the brain;	Vildagliptin exhibited improvement memory retention and attenuation of Aβ, tau phosphorylation and inflammatory markers and increased GLP-1 level.
			iii: ↑ cresyl violet-positive neurons in the brain;	
			v: ↑ learning and memory: radial arm maze task; hole-board task;	
Kosaraju et al. (2017)	Linagliptin	The 3xTg-AD mouse model of AD received linagliptin orally (5, 10, and 20 mg/kg) for 8 weeks.	i: ↑ the level of GLP-1 and GIP in the brain but had no effect on plasma glucose level;	Linagliptin, a dipeptidyl peptidase-4 inhibitor, mitigates cognitive deficits and pathology in the 3xTg-AD mouse model of AD.
			ii: ↓ Aβ_42_ in the brain; ↓ p-tau in the brain; ↓ neuroinflammation;	
			iii: ↓ thioflavin S positive plaques in the brain;	
			v: ↑ cognitive performance: MWM; Y-maze;	
Babic et al. (2018)	Liraglutide	SD rats were randomly treated with olanzapine (2 mg, tid), clozapine (12 mg/kg, tid), liraglutide (0.2 mg/kg, tid), olanzapine + liraglutide co-treatment, clozapine + liraglutide co-treatment or vehicle for 6 weeks.	i: ↑ clozapine-induced glucose intolerance; ↓ olanzapine-induced weight gain, adiposity;	Liraglutide co-treatment improved aspects of cognition, prevented obesity side effects of olanzapine, and the hyperglycemia caused by clozapine.
			v: prevented olanzapine- and clozapine-induced deficits in recognition memory: ↓ the NOR test discrimination ratio; partially reversed olanzapine-induced working memory (T-Maze test) and voluntary locomotor activity deficits;	
Jantrapirom et al. (2020)	Liraglutide	SH-SY5Y cells incubated with or without high level of insulin (100 nM) for 48 h before treatment with liraglutide (500 nM) for 24 h.	ii: ↑ the phosphorylation of IR, IRS-1, Akt and GSK3β; ↓ the formation of Alzheimer’s markers and plaque (amyloid plaque and tau phosphorylation); ↓ BACE-1 activity in insulin resistant neurons;	Liraglutide restored neuronal insulin resistance and ameliorated AD markers.
Gad et al. (2016)	Pioglitazone and exenatide	Male Wistar albino rats received fructose to induce insulin resistance before treatment with pioglitazone (10 mg/kg), exenatide (10 or 20 μg/kg), pioglitazone plus exenatide or vehicle for 8 weeks.	Monotherapy or combination of pioglitazone and exenatide	The combination of pioglitazone and exenatide offered hippocampal neuroprotection and produced pro-cognitive effect in insulin resistant rats.
			i: ↓ blood glucose levels, insulin level and HOMA-IR index; ↓ serum advanced glycated end products; ↓ serum lipids: TG, TG, LDL levels;	
			iii: ↓ percent of hippocampal pycnotic cells; ↓ hippocampal Aβ expression; ↓ hippocampal microglia expression;	
			v: ↑ cognition: eight-arm radial maze test;	
Chen et al. (2015)	Pioglitazone	12-month-old APP/PS1 mice received pioglitazone i.p. (10 mg/kg/day) for 15 days.	In AD mouse model:	Pioglitazone inhibited Cdk5 activity by decreasing p35 protein level and rescued impaired synaptic plasticity and spatial memory in AD mouse models.
			ii: ↓ Cdk5 activity by decreasing p35 protein level;	
			iii: reversed Aβ-induced dendritic spine loss;	
			iv: rescued LTP defects;	
			v: ↑ spatial memory: MWM;	
Yu et al. (2015)	pioglitazone or rosiglitazone	3xTg-AD mice and wild type control mice received an experimental diet containing pioglitazone hydrochloride or rosiglitazone maleate for 4 months.	In 3xTg-AD mice	The chronic treatment of 3xTg-AD mice with pioglitazone or rosiglitazone for 4 months improved spatial learning and attenuated tau hyperphosphorylation and neuroinflammation.
			i: pioglitazone and rosiglitazone: ↓ the body weight;	
			iii: pioglitazone and rosiglitazone: ↓ tau phosphorylation in the hippocampus; ↑ AKT signaling in the brain; ↓ neuroinflammation;	
			v: pioglitazone improved learning ability: MWM;	
Escribano et al. (2010)	Rosiglitazone	9-month-old transgenic mice overexpressing human amyloid precursor protein (hAPP) received rosiglitazone p.o. 5 mg/kg/day for 4 weeks.	ii: ↓ brain Aβ levels and Aβ plaque deposition; ↓ p-Tau aggregates;	Rosiglitazone reduced AD pathology and restored hippocampal function, leading to a rescue of memory impairment in APP transgenic mice.
			v: ↓ memory deficits: object recognition and MWM;	
Sabogal-Guáqueta et al. (2015)	Quercetin	3xTg-AD mice received quercetin (25 mg/kg i.p.) or vehicle every 48 hours for 3 months.	ii: ↓ β-amyloidosis, βA 1–40 and βA 1–42 in the brain; ↓tauopathy in the brain; ↓ astrogliosis and microgliosis in the brain;	Quercetin ameliorated cognitive deficits, reversed brain levels of β-amyloidosis and tauopathy and ameliorated astroglia and microglia reactivity in the 3xTg-AD mice.
			iii: ↑ the cell density in the subiculum;	
			v: ↑ spatial learning and memory performance: MWM; exerted anxiolytic effect: EPM tests	
Wang et al. (2014)	Quercetin	APPswe/PS1dE9 mice received quercetin (20, 40 mg/kg bw, once daily) or Aricept (2 mg/kg once daily) or vehicle for 16 weeks.	ii: ↓ plaque pathology; attenuated mitochondrial damage: mitochondrial membrane potential, ATP levels; ↓ ROS production; ↑ AMPK activity;	Quercetin ameliorated cognitive deficits, reduced sensile plaques, and ameliorated mitochondrial dysfunction.
			v: ↑ recognition memory, learning and memory function: novel object recognition and MWM;	
Sriraksa et al. (2012)	Quercetin	Male Wistar rats received quercetin (100, 200, 300 mg/kg bw, once daily) orally or vehicle or levodopa or vitamin C for 14 days before and 14 days after the unilateral lesion of right substantia nigra induced by 6-OHDA.	ii: ↓ Ache activity (300 mg/kg); ↓ MDA levels (300 mg/kg); ↑ SOD, CAT and GPx activity in the hippocampus (300 mg/kg);	Quercetin enhanced spatial memory partly because of decreased oxidative damage resulting in decreased neuron density.
			iii: ↑ density of survival neuron in the hippocampus;	
			v: ↑ learning and memory: MWM;	
Mehta et al. (2017)	Quercetin	Swiss albino mice were subjected to an array of unpredicted stressors for 21 days during which 30 mg/kg quercetin treatment was given orally.	i: normalized chronic unpredicted stressors mediated elevated blood glucose level, elevated serum corticosterone level, serum insulin and insulin sensitivity;	Quercetin improved chronic unpredicted stressors mediated cognitive dysfunction by modulating hippocampal insulin signaling.
			ii: ↑ the expression of IR and GLUT4;	
			iii: alleviated chronic unpredicted stressors mediated neuronal damage in hippocampus;	
			v: alleviated chronic unpredicted stressors mediated cognitive dysfunction: NOR, MWM;	
Mert et al. (2019)	Quercetin	40 Balb-C received corn oil + saline or quercetin 50 mg/kg/day + saline or corn oil + ketamine or quercetin 25 mg/kg/day+ ketamine or quercetin 50 mg/kg/day + ketamine for 21 days.	ii: quercetin (50 mg/kg) ↓ the level of MDA ↑ the levels of GPx and SOD in both the hippocampus and prefrontal cortex in ketamine-administered mice;	Quercetin improved ketamine induced cognitive deficits in mice partly owing to its ability to scavenge free radicals and its high antioxidant capacity.
			v: improved ketamine induced cognitive deficits;	
Shirai et al. (2012)	Sulforaphane	Male Std: ddy mice received sulforaphane (3, 10 and 30 mg/kg, i.p.) after administration of PCP (3 mg/kg, s.c.).	v: Sulforaphane (30 mg/kg, i.p.) attenuated hyperlocomotion in mice after PCP administration;	Sulforaphane attenuated hyperlocomotion and PPI deficits in mice after PCP administration in a dose-dependent manner.
			Sulforaphane (3, 10 and 30 mg/kg, i.p.) attenuated PPI deficits in mice after PCP administration;	
Shirai et al. (2015)	Sulforaphane	Schedule 1: male ICR mice received vehicle + saline, or sulforaphane (30 mg/kg/day, i.p.) + saline, or vehicle + PCP (10 mg/kg/day, s.c.) or sulforaphane (30 mg/kg/day, i.p.) + PCP (10 mg/kg/day, s.c.) for 10 days. Schedule 2: After schedule 1, sulforaphane (30 mg/kg/day, i.p.) or the vehicle was administered once daily for 14 days.	iii: schedule 1: pretreatment with sulforaphane attenuated PCP-induced reduction in the spine density, protected against the PCP-induced increase in the 8-oxo-dG-positive cells and decrease in PV-positive cells in the mPFC and hippocampus;	Sulforaphane had prophylactic and therapeutic effects on PCP-induced cognitive deficits in mice.
			v: NOR: schedule 1: pretreatment with sulforaphane attenuated PCP-induced cognitive deficits in mice;	
			schedule 2: sulforaphane attenuated PCP-induced cognitive deficits in mice;	

IGF1R: type 1 insulin-like growth factor receptor; PDK1: 3-phosphoinositide-dependent protein kinase-1; 2-DOG: [3H]2-deoxyglucose; AMPK: AMP activated protein kinase; IR: insulin receptor; IRS: insulin receptor substrate; PI3K: Phosphatidyl inositol 3-kinase; Akt: protein kinase B; GSK3: glycogen synthase kinase 3; ERK: extracellular regulated kinase; FAK: focal adhesion kinase; Ache: Acetylcholinesterase; NF-κB: nuclear factor κB; ROS: reactive oxygen species; OFT: Open Field Test; FST: forced Swim Test; EPM: Elevated Plus Maze; TST: Tail Suspension Test; MWM: Morris Water Maze; SOD: superoxide dismutase; CAT: catalase; GPx: superoxide dismutase; GR: glutathione reductase; TNF-α: tumor necrosis factor-alpha; IL-1β: interleukine-1beta; CREB: cAMP response element binding protein; BDNF: brain-derived neurotrophic factor; PPI: pre-pulse intensity; ROS: reactive oxygen species; EGFR: Epidermal Growth Factor Receptor; Sirt1: silent information regulator 2 homolog 1; BACE-1: Beta Secretase 1; PS1: presenilin1; APP: amyloid precursor protein; NOR: Novel Object Recognition; RAGE: receptor for advanced glycation end products; ICV: intracerebroventricular; LA: L-arginine; L-NAME: nitro-L-arginine methyl ester; ORT: the object recognition test; STZ: streptozotocin; TrkB: tropomyosin-related kinase B receptor; p75NTR: p75 neurotrophin receptor; PAL: The passive avoidance learning; Mash1: Mammalian achaete-scute homologue 1; GIPR: gastric inhibitory polypeptide receptor; VEGF: vascular endothelial growth factor; T-AOC: total antioxidant capability; GSH: glutathione; GSHPx: Glutathione peroxidase; PCP: phencyclidine; RNS: reactive nitrogen species; Cdk5: Cyclin-dependent kinase 5.

**TABLE 2:** Clinical studies: targeting insulin pathway to improve cognitive deficits

Refs	Diagnosis	Design	Intervention(n); Control(n)	Drug, duration	Results
Benedict et al. (2004)	Healthy subjects	Randomized, double-blind, placebo-controlled	intranasal insulin 40 IU (19);	Intranasal insulin 40 IU;	Intranasal intake of insulin enhanced both consolidation of words and general mood in humans without causing systemic side effects.
			placebo (19)	8-week treatment	
Craft et al. (2012)	Adults with amnestic mild cognitive impairment or AD	Randomized, double-blind, placebo-controlled	intranasal insulin 20 IU (36);	Intranasal insulin 20 IU or 40 IU; 4-month treatment	Treatment with 20 IU of insulin improved delayed memory. Both doses of insulin (20 and 40 IU) preserved caregiver-rated functional ability and general cognition (the ADAS-cog score and the ADCS-ADL scale).
			intranasal insulin 40 IU (38);		
			placebo (30)		
Claxton et al. (2015)	Adults with amnestic mild cognitive impairment or AD	Randomized, double-blind, placebo-controlled	intranasal insulin 20 IU (21);	Intranasal insulin 20 IU or 40 IU; 3-week treatment	Daily treatment with 40 IU insulin modulated cognition for adults with AD or mild cognitive impairment, with the apolipoprotein E-related differences in treatment response for the primary memory composite.
			intranasal insulin 40 IU (19);		
			placebo (20)		
Craft et al. (2020)	Adults with amnestic mild cognitive impairment or AD	Randomized, double-blind, placebo-controlled	intranasal insulin 40 IU (119);	Intranasal insulin 40 IU; 12-month treatment; Followed by a 6-month open label extension	No cognitive or functional benefits were observed with intranasal insulin treatment over a 12-month period among the primary intention-to-treat cohort.
			placebo (121);		
Cha et al. (2017)	Major depressive disorder	Randomized, double-blind, placebo-controlled crossover design	intranasal insulin 40 IU (19);	intranasal insulin 40 IU; 4 times/d;	No between group differences were observed in change from baseline on total MADRS, PANAS, or on a global index of neurocognition.
			placebo (16)	12-week treatment	
McIntyre et al. (2012)	Euthymic adults with bipolar disorder	Randomized, double-blind, placebo-controlled	intranasal insulin 40 IU (34);	Intranasal insulin 40 IU qid;	Adjunctive intranasal insulin administration significantly improved a single measure of executive function in bipolar disorder.
			placebo (28)	8-week treatment	
Fan et al. (2011)	Nondiabetic patients with SZ or schizoaffective disorders	Single dose, double-blind, placebo-controlled	single-dose of intranasal insulin 40 IU (15);	single dose of intranasal insulin 40 IU	Single-dose intranasal insulin treatment did not have a large-enough effect on verbal memory or sustained attention to be detected in this study.
			placebo (15)		
Fan et al. (2013)	SZ or schizoaffective disorders	Randomized, double-blind, placebo-controlled	intranasal insulin 40 IU 4 times/d (21);	Adjunctive intranasal insulin 40 IU 4 times/d; 8-week treatment	There were no significant differences between the two groups at week 8 on psychopathology and cognition (PANSS and SANS).
			placebo (24)		
Guo et al. (2014)	Depression with T2DM	Randomized, double-blind, placebo-controlled	metformin (29);	Metformin 1000 mg once-daily for 1 week, then daily dose increased to 1500 mg over a period of 2 weeks (tid); 24-week treatment	Metformin changed glucose metabolism (HbA1c levels), improved depressive performance (MADRS and HRSD-17 scores) and cognitive function (Wechsler Memory Scale–Revised).
			placebo (29)		
Luchsinger et al. (2016)	Amnestic mild cognitive impairment	Randomized, double-blind, placebo-controlled	metformin (40);	Metformin, (or placebo) 1000 mg (bid);	Metformin improved cognitive deficits (total recall of the Selective Reminding Test).
			placebo (40)	12-month treatment	
Koenig et al. (2017)	Non-diabetic subjects with mild cognitive impairment and early dementia due to AD	Randomized, double-blind, placebo-controlled, crossover study	placebo followed by metformin (10);	Metformin 500 mg (or placebo) by mouth daily for 1 week, then daily dose increased to 2000 mg (bid);	Metformin improved executive function (Trails-B) and increased orbitofrontal metabolism (Arterial Spin Label MRI).
			metformin followed by placebo (10)	16-week treatment (8 weeks metformin followed by placebo for 8 weeks or vice versa)	
Lin et al. (2018)	NDVCI	Randomized, double-blind	metformin + donepezil (48);	Metformin, 500 mg (tid); Donepezil 10 mg (qn); Acarbose, 50 mg (tid); 1-year treatment	Metformin improved cognitive function (ADAS-Cog scores; WHO-UCLA AVLT TMT scores; TMT times).
			acarbose + donepezil (46)		
Mansur et al. (2017)	Mood disorder (MDD or BD)	Open-label	19	Liraglutide, 1.8 mg, daily;	Liraglutide improved cognitive function from baseline to endpoint (the TMTB standard score and a composite Z-score comprising multiple cognitive tests (the DSST, RAVLT, Stroop test))
				4-week treatment	
Ishoy et al. (2017)	SZ	Randomized, double-blind, placebo-controlled	exenatide (20);	Exenatide, 2 mg once weekly;	Non-significant result in improving cognition or psychosocial function in obese, antipsychotic-treated SZ patients (BACS, REY, SF-36, PSP, PANSS)
			placebo (20)	3-month treatment	
Watson et al. (2019)	Subjective cognitive complaints (half of subjects had family history of AD; 45-70 years old)	Randomized, double-blind, placebo-controlled	liraglutide (15);	Liraglutide 0.6 mg for 1 week, 1.2 mg for 1 week, 1.8 mg for 10 weeks, daily;	No detectable cognitive differences between study groups after this duration of treatment.
			placebo (11)	12-week treatment	
Watson et al. (2005)	Mild AD or amnestic mild cognitive deficits	Randomized, double-blind, placebo-controlled	rosiglitazone (20);	Rosiglitazone, 4 mg, daily;	Rosiglitazone exhibited better delayed recall and selective attention compared to placebo.
			placebo (10)	6-month treatment	
Hanyu et al. (2009)	AD or amnestic mild cognitive deficits	Randomized, open-controlled	pioglitazone (15);	Pioglitazone, 15 to 30 mg, daily;	Pioglitazone decreased FIRI and HOMA-R, and improved cognition (ADAS-Jcog scores and WMS-R logical memory-I scores).
			control (17)	6-month treatment	
Sato et al. (2011)	AD with T2DM	Randomized, open-controlled	pioglitazone (21);	Pioglitazone, 15-30 mg, daily (15 mg *n*=19; 30 mg *n*=2);	Pioglitazone decreased plasma insulin levels, improved cognition (MMSE, ADAS-J-cog, and WMS-R logical memory-I) and regional cerebral blood flow in the parietal lobe.
			control (21)	6-month treatment	
Yi et al. (2012)	SZ	Randomized, double-blind, placebo-controlled	rosiglitazone (9);	Rosiglitazone, 4 mg, daily;	No significant results in change scores of cognitive performances between two groups in clozapine-treated patients with SZ (WAIS-III, HVLT, TMT, WCST)
			placebo (10)	8-week treatment	
Broman-Fulks et al. (2012)	Healthy subjects	Randomized, double-blind, placebo-controlled	Quercetin 500 mg/day (309);	Quercetin 500 mg/day;	No significant effects of quercetin on memory, psychomotor speed, reaction time, attention or cognitive flexibility between groups.
			Quercetin 1000 mg/day (319);	Quercetin 1000 mg/day;	
			Placebo (313);	12-week treatment	
Shiina et al. (2015)	SZ	Open-label, preliminary clinical trial	Sulforaphane (7)	Sulforaphane (30 mg/day p.o); 8-week treatment	Sulforaphane had the potential to improve some domains of cognitive function in SZ (OCLT).

NDVCI: non-dementia vascular cognitive impairment; ADAS-Cog: the Alzheimer’s Disease Assessment Scale-Cognitive Subscale; WHO–UCLA AVLT: The World Health Organization–University of California–Los Angeles Auditory Verbal Learning Test; TMT: the Trail Making Test; SZ: schizophrenia; MDD: major depressive disorder; BD: bipolar disorder; TMTB: the Trail Making Test-B; DSST: Digit Symbol Substitution Test; RAVLT: Rey Auditory Verbal Learning Test; BACS: Brief Assessment of Cognition in Schizophrenia; REY: Rey-Osterreith complex figure test; SF-36: the Short-Form 36 survey of the International Quality of Life Assessment; PSP: the Personal and Social Performance Scale; PANSS: Positive and Negative Syndrome Scale; SANS: the Scale for Assessment of Negative Symptoms; WAIS-III: the Digit Span subtest from the Wechsler Adult Intelligence Scale III; HVLT: the verbal fluency test, the Hopkins Verbal Learning Test; TMT: the Trail-Making Test; WCST: the Wisconsin Card Sorting Test; MMSE: the Mini-Mental State Examination; ADAS-J-cog: Alzheimer’s Disease Assessment Scale-Cognitive Subscale Japanese version; ADCS-ADL: The Alzheimer’s Disease Cooperative Study– activities of daily living; WMS-R logical memory-I: Wechsler Memory Scale-revised logical memory-I; FIRI: fasting immunoreactive insulin; HOMA-R: the homeostasis model assessment ratio; OCLT: the Accuracy component of the One Card Learning Task.

### Intranasal Insulin

The reductions in the integrity of blood brain barrier may also be a contributing factor to the pathologic cascade leading to cognitive decline in neurodegenerative and neuropsychiatric disorders(Haar et al., 2016). Under the circumstances, intranasal insulin provides an advantageous and non-invasive technique to directly supply the brain with insulin to better target and bypass the blood brain barrier without significant influences on plasma insulin or glucose levels(Renner et al., 2012). Furthermore, intranasal insulin had a faster time course of absorption compared with subcutaneous insulin and the bioavailability of intranasal insulin was 8.3% compared with an intravenous bolus injection(Drejer et al., 1992). Studies demonstrated that intranasal insulin could improve memory function in cognitively impaired humans(Freiherr et al., 2013), providing the possibility that intranasal insulin was potentially a promising strategy to treat cognitive deficits.

#### Preclinical Studies

Fourteen consecutive days of intranasal insulin treatment could prevent the severe memory and learning impairment induced by intracerebroventricular administration of Aβ, which was a rat model of AD(Farzampour et al., 2016). In transgenic mouse model of AD, intranasal insulin improved cognitive impairment through promotion of neurogenesis, alleviation of Aβ pathology and enhancement of insulin signaling(Mao et al., 2016). Intranasal delivery of insulin for 7 days restored insulin signaling, increased synaptic protein expression and reduced Aβ levels and microglial activation in the brains of AD mouse model(Chen et al., 2014). These studies suggested that the beneficial effects of intranasal insulin on cognitive deficits could be partially due to the enhancement of insulin signaling and synaptic plasticity and the reduction of Aβ level and neuroinflammation in the brain.

#### Clinical Studies

In healthy subjects, intranasal intake of insulin improved consolidation of words and general mood, indicating intranasal insulin as a potential treatment in patients showing cognitive deficits in conjunction with a lack of insulin(Benedict et al., 2004). Acute administration of intranasal insulin enhanced functional connectivity between the dorsal medial prefrontal cortex of the default-mode network and the hippocampus in healthy lean, overweight and obese adults, although this study missed examination on cognitive function(Kullmann et al., 2017).

Previous research studies have evaluated the cognitive beneficial effect of intranasal insulin therapy in non-schizophrenia patients. Adjunctive intranasal insulin administration was safe, well tolerated, and effective on a measure of executive function in bipolar disorder(McIntyre et al., 2012). Systematic reviews also suggested that intranasal insulin administration might have a beneficial effect on cognitive function in amnestic mild cognitive impairment and AD(Lu and Xu, 2019), probably modified by apolipoprotein (APOE) 4 allele carrier status(Avgerinos et al., 2018). A pilot clinical trial demonstrated that 4 months intranasal insulin treatment stabilized or improved cognition, function and cerebral glucose metabolism for adults with amnestic mild cognitive impairment and AD(Craft et al., 2012). Other studies showed sex and APOE genotype differences in treatment response to different doses of intranasal insulin in adults with mild cognitive impairment or AD(Reger et al., 2008; Claxton et al., 2013). There may be a fundamental difference in central insulin sensitivity in sex and APOE genotype differences(Claxton et al., 2013). Long acting intranasal insulin detemir also provided cognitive benefit for individuals diagnosed with mild cognitive impairment and AD dementia, and in particular for memory-impaired adults who were APOE-Ɛ4 carriers(Claxton et al., 2015). More recently, a randomized clinical trial demonstrated that insulin modulated aspects of brain function relevant to AD and could be delivered into the brain using intranasal devices, although this study failed to confirm the cognitive benefit of intranasal insulin(Craft et al., 2020). A clinical trial did not demonstrate statistically significant improvements on overall mood, aspects of emotional processing, neurocognitive function, or self-reported quality of life patient reported outcomes in major depressive disorder either(Cha et al., 2017).

The cognitive benefit of intranasal insulin in healthy subjects and AD may not generalize to schizophrenia. Single-dose intranasal insulin treatment (40 IU) did not have significant beneficial effect on verbal memory and sustained attention in patients with schizophrenia(Fan et al., 2011). This trial excluded diabetic patients and the negative findings might be explained by the small sample size, the dosing of insulin, the severity of baseline cognitive deficits in study subjects and the timing of posttreatment cognitive assessment(Fan et al., 2011). Another 8-week clinical trial also failed to demonstrate any significant beneficial effect of intranasal insulin on cognition in patients with schizophrenia(Fan et al., 2013). Likewise, there were no significant differences in body metabolism between the adjunctive therapy of intranasal insulin (40 IU 4 times per day) group and the placebo group for schizophrenia patients(Li et al., 2013). However, these studies excluded diabetes mellitus subjects and it was possible that the adjunctive intranasal insulin treatment was not able to mitigate the side effect of antipsychotic treatment that patients received before(Fan et al., 2013; Li et al., 2013). Another challenge is the uncertainty of how efficient the drug is delivered into the brain via intranasal insulin. Larger sample size and multi-site studies considering dose, course and nasal-to-brain insulin delivery efficiency are expected to investigate whether intranasal insulin treatment shows beneficial effect in schizophrenia patients with cognitive deficits.

### Metformin

Metformin, a biguanide anti-diabetic drug, is recommended as first line treatment in T2DM. The main effect of metformin is to acutely decrease hepatic glucose production through the inhibition of mitochondrial respiratory chain complex Ⅰ and subsequent the activation of AMPK(Viollet et al., 2011). Metformin is also known to have numerous nonglycemic effects. Metformin could activate AMPK pathway to protect neurons from oxidative injury(Zhao et al., 2019), produce antidepressant effects(Fang et al., 2020), and prevent neuroinflammation and neurodegeneration (Paudel et al., 2020).

#### Preclinical Studies

Chronic hyperinsulinemia in Neuro2a cells leaded to reduction of phosphorylation of IRS-1, PI3K, translocation of glucose transporter (GLUT) 4 and expression of GLUT3, and metformin could directly reverse such neuron insulin resistance status(Gupta et al., 2011). Metformin protected rats against from methamphetamine-induced neurodegeneration via the modulation of Akt/GSK3 or CREB/BDNF signaling pathway, and the amelioration of behavioral changes such as anxiety, depression and cognitive deficits(Keshavarzi et al., 2019). Metformin attenuated spatial memory deficits, neuron loss in the hippocampus, decreased Aβ plaque load and chronic inflammation in the hippocampus and cortex, and enhanced neurogenesis in the APP/PS1 mice, mouse model of AD(Ou et al., 2018; Chen et al., 2021). Metformin also restored spine density, surface AMPA subunit GluA1 trafficking, LTP expression and spatial memory in the APP/PS1 mouse by inhibiting cyclin-dependent kinase 5 hyper-activation and cyclin-dependent kinase 5-dependent tau hyperactivation(Wang et al., 2020). Furthermore, metformin could activate AMPK and insulin-degrading enzyme in the brain of APP/PS 1 mice, which might be the key neuroprotection mechanism of metformin(Lu et al., 2020). Metformin treatment for 8 weeks improved memory in the SAMP8 mouse model of sporadic AD, which was associated with the decreased levels of APPc99 and pTau404(Farr et al., 2019). In MK801-induced schizophrenia-like rats, metformin attenuated the olanzapine- and risperidone-induced metabolic dysfunctions without reducing the therapeutic effects of the antipsychotics(Luo et al., 2020). Furthermore, in MK-801-induced schizophrenia-like models, metformin ameliorated the pre-pulse inhibition deficits, alleviated hyperactivity, the anxiety-like behaviors, recognition and spatial memory impairment through normalizing the phosphorylation of Akt and GSK3β in the cerebral cortex of rats(Wang et al., 2019).

#### Clinical Studies

At present, the relationship between metformin and cognitive performance is controversial in clinical studies. In patients with non-dementia vascular cognitive impairment and abnormal glucose metabolism, a year metformin treatment was effective in improving cognitive function, especially in terms of performance function(Lin et al., 2018). Metformin also produced cognitive and glucose metabolism improvement among depressed patients with diabetes mellitus(Guo et al., 2014). In patients with amnestic mild cognitive impairment or non-diabetes subjects with mild cognitive impairment or mild dementia due to AD, metformin treatment was associated with promising cognitive enhancement(Luchsinger et al., 2016; Koenig et al., 2017). However, a population-based Mayo clinic study of aging observed that metformin was associated with an increased risk of mild cognitive impairment and was not associated with cognitive test performance(Wennberg et al., 2018). In schizophrenia, evidence suggested that metformin treatment resulted in significant reduction in weight gain and insulin resistance in patients treated with antipsychotics(Wu et al., 2008a; Wu et al., 2008b; de Silva et al., 2016). A clinical trial recruited 120 participants to investigate the impact and the related mechanism of metformin treatment on cognitive deficits of schizophrenia co-morbid metabolic syndrome (clinicaltrials.gov: NCT03271866). More longitudinal studies are needed to verify the cognitive outcomes of metformin and more works are expected to investigate whether metformin treatment is effective in enhancing cognitive performance in schizophrenia patients.

### GLP-1 Receptor Agonists

GLP-1, an incretin hormone from the enteroendocrine L-cells, increases insulin secretion and reduces glucose excursion and glucagon secretion to regulate glucose metabolism. Endogenous GLP-1 is rapidly degraded by dipeptidyl peptidase-4 (DPP-4) enzyme. Therefore, the DPP-4 inhibitor (vildagliptin or sitagliptin) and the DPP-4 resistant GLP-1 analogues (exenatide, liraglutide, dulaglutide and lixisenatide) are developed to overcome the short hart-life of endogenous GLP-1(Sharma et al., 2018). The activation of GLP-1 receptors in periphery and central nervous system both mediate GLP-1’s food intake inhibitory and glycemic effects(Hayes, 2012). Additionally, the activation of GLP-1 receptors in the brain related to cognition exhibits neuroprotection effects, involving neuronal excitability, survival and proliferation(Mansur et al., 2018). The DPP-4 inhibitors, vildagliptin or sitagliptin, also exhibited cognitive enhancement effects(Pipatpiboon et al., 2013; Gault et al., 2015).

#### Preclinical Studies

In preclinical studies, GLP-1 receptor agonists protected PC12 cells from apoptosis involving EGFR transactivation and subsequent activation of the PI3K/Akt/mTOR signaling pathway(Kimura et al., 2013), and glucose metabolic aberration involving Sirt1-dependent deacetylation and Akt-dependent phosphorylation of Forkhead box O (FoxO) 1(Chen et al., 2019a). Liraglutide could reverse deleterious effects of insulin resistance in neuronal cells by improving the phosphorylation status of insulin receptor, IRS-1 and Akt(Jantrapirom et al., 2020). Exendine-4, a long-acting GLP-1 receptor agonist, inhibited glucose-induced apoptosis and oxidative stress in neurons(Chen et al., 2012), and ameliorated cognitive impairment(Chen et al., 2012) involving its metabolic, anti-inflammatory and anti-oxidant effects(Solmaz et al., 2015; Abdelwahed et al., 2018). The activation of GLP-1 receptor could attenuate neuroinflammation and improve neurogenesis and insulin sensitivity in AD(Bae and Song, 2017). For example, dulaglutide, a novel GLP-1 receptor agonist, ameliorated STZ induced AD-like impairment of learning and memory ability by modulating hyperphosphorylation of tau and neurofilaments through improving the PI3K/AKT/GSK3β signaling pathway(Zhou et al., 2019). Liraglutide reversed cognitive impairment in mice and attenuated insulin receptor and synaptic pathology in a non-human primate model of AD(Batista et al., 2018). Specifically, in mice, 7 days liraglutide treatment prevented the loss of brain insulin receptors and synapses, and reversed memory impairment caused by intracerebroventricular administration of Aβ oligomers, while systemic treatment on non-human primates with liraglutide indicated partial neuroprotection, decreasing AD-related insulin receptor, synaptic, and tau pathology in specific brain regions(Batista et al., 2018). Moreover, in AD mouse models, administration of liraglutide could reduce Aβ plaques, decrease tau hyperphosphorylation and neurofilament proteins, ameliorate chronic inflammation, attenuate oxidative/nitrosative stress, and improve cognitive function (McClean et al., 2011; Xiong et al., 2013; Hansen et al., 2015; McClean et al., 2015; Duarte et al., 2020). The combination of exenatide and insulin was associated with better memory and normal expression of insulin receptor pathway genes in a mouse model of AD(Robinson et al., 2019). And exenatide could improve cognitive decline through inhibiting apoptosis and promoting the BDNF-TrkB neurotrophic axis in adult wild-type mice(Bomba et al., 2018). Exenatide was reported to improve cognitive impairment by reducing Aβ_1-42_ deposition, alleviating synaptic degradation, and improving hippocampal mitochondrial morphology and dynamics in the mouse model of AD(An et al., 2019). However, another study suggested that exenatide could enhance BDNF signaling and reduce inflammation in AD mouse model, but it failed to promote changes in cognitive function(Bomba et al., 2019). DPP-4 inhibitors, sitagliptin and saxagliptin, also protected learning and memory function in AD mouse model through increasing the O-Glycosylation and decreasing abnormal phosphorylation of tau and neurofilaments (NFs), reducing intercellular Aβ accumulation and alleviating neurodegeneration related to GLP-1 signaling pathway(Chen et al., 2019b). Additionally, saxagliptin and vildagliptin reduced inflammation in streptozotocin-induced AD mice(Kosaraju et al., 2013a; Kosaraju et al., 2013b). Linagliptin mitigated cognitive deficits, improved brain incretin levels and attenuated Aβ, tau phosphorylation as well as neuroinflammation in mouse model of AD(Kosaraju et al., 2017). In Sprague–Dawley rats undergoing antipsychotics treatment, liraglutide ameliorated glucose metabolism dysregulation and improved cognitive deficits(Babic et al., 2018). In specific, from the start of antipsychotics treatment, liraglutide co-treatment prevented olanzapine-induced working and recognition memory deficit and obesity side-effect, and clozapine-induced reductions in recognition memory and hyperglycemia(Babic et al., 2018).

#### Clinical Studies

A systematic review demonstrated that GLP-1 receptor agonists induced a weight loss and improved fasting glucose and HbA1c in schizophrenia patients on treatment who were overweight or obese(Siskind et al., 2019). Treatment with liraglutide once-daily subcutaneous injection for 16 weeks improved glucose tolerance and weight gain in clozapine- and olanzapine-treated schizophrenia patients who were overweight and prediabetes compared with controls(Larsen et al., 2017). And liraglutide exhibited pro-cognitive effects in individuals with mood disorder in a 4-week, open-label study(Mansur et al., 2017).While 3 months treatment with exenatide 2 mg once weekly failed to improve cognition in obese antipsychotics-treated schizophrenia patients(Ishoy et al., 2017). However, the study excluded patients with diabetes and the dosage, duration and statistical power may contribute to the negative results(Ishoy et al., 2017). In mid-life individuals at risk of AD, there was an inverse correlation between insulin resistance and connectivity between bilateral hippocampal and anterior medial frontal structures(Watson et al., 2019). However, the enhancement in connectivity after liraglutide treatment was not accompanied with improved cognitive function(Watson et al., 2019). It is necessary to consider disease differences, sample size and duration of treatment when explore the effects of GLP-1 receptor agonists in cognitive function.

### Peroxisome Proliferator-Activated Receptors (PPAR) γ Agonists

PPAR γ is a member of the nuclear receptor family that regulates gene expression by binding to DNA sequence elements termed PPAR response elements, involving in adipocyte biology, insulin action, cardiovascular disease, inflammation, renal function, and tumor biology(Lehrke and Lazar, 2005). Specific PPAR γ ligands are divided into two major groups: endogenous or natural agonists and synthetic agonists. Evidence suggested that Thiazolidinediones, synthetic high PPAR γ-selective ligands, may constitute a potentially novel and innovative treatment approach for cognitive deficits by means of salutary effects on altered inflammatory and metabolic networks(McIntyre et al., 2007). PPAR γ agonists may be a possible therapeutic target in neuropathological conditions, showing anti-inflammatory and anti-oxidation effects, protective action on cerebral glucose and glutamate metabolism and inducing neuronal growth factor and BDNF production(Garcia-Bueno et al., 2010).

#### Preclinical Studies

In neuronal seipin knock-out mice, rosiglitazone rescued the hippocampal LTP induction and ameliorated spatial cognitive deficits(Zhou et al., 2016). Pioglitazone also exhibited learning and memory improvement in alcohol-induced neuronal damage rats(Cippitelli et al., 2017). Monotherapy or combination of pioglitazone and exenatide improved cognitive function, decreased hippocampal neurodegeneration and reduced microglia overexpression in insulin resistant rats(Gad et al., 2016). In a mouse model of AD, pioglitazone and fenofibrate combined treatment ameliorated memory and cognitive impairment via modulation of the Wnt/beta catenin pathway, accompanied with a reduction in neuronal damage(Assaf et al., 2020). Pioglitazone could rescue impaired synaptic deficits and spatial memory in AD transgenic mice through inhibiting cyclin-dependent kinase 5 activity and reversing Aβ-induced dendritic spine loss(Chen et al., 2015). The chronic treatment of AD transgenic mice with pioglitazone or rosiglitazone for 4 months improved spatial learning, enhanced AKT signaling and attenuated tau hyperphosphorylation and neuroinflammation(Yu et al., 2015). Rosiglitazone rescued AD pathology and restored the hippocampal function, leading to a rescue of memory impairment in 9-month-old AD transgenic mice(Escribano et al., 2010). Rosiglitazone showed cognitive improvement through multiple ways such as regulating extracellular signal-regulated protein kinase MAPK signaling transduction(Denner et al., 2012), and affecting neuronal ion channel and synaptic plasticity in the brain of Tg2576(Nenov et al., 2014; Hsu et al., 2017), an extensively characterized AD mouse model.

#### Clinical Studies

Anti-diabetic agents, PPAR γ agonists, might have the pro-cognitive effects in subjects with AD/mild cognitive impairment with good acceptability(Cao et al., 2018). In patients with mild AD or amnestic mild cognitive deficits, 6-months treatment of rosiglitazone exhibited cognitive improvement compared with placebo(Watson et al., 2005). Pioglitazone also ameliorated glucose metabolism and cognitive function in AD(Hanyu et al., 2009; Sato et al., 2011). However, a systematic review including nine eligible studies suggested that there was insufficient evidence to support the cognitive benefits of rosiglitazone in AD and amnestic mild cognitive impairment patients although the efficacy of pioglitazone was promising in patients with comorbid diabetes(Liu et al., 2015). A study demonstrated that among antipsychotics-treated patients with schizophrenia, there was a significant association of PPAR γ gene in altered glucose levels and psychosis profile(Liu et al., 2014). Arulmozhi suggested a possible role of PPAR agonists in antipsychotic-induced insulin resistance rodents(Arulmozhi et al., 2006), and such medications were related with BDNF levels in schizophrenia, which might play a part in the management of this illness(d'Angelo et al., 2019). In a small pilot clinical trial, rosiglitazone treatment for 8 weeks failed to show the potential cognitive benefit in schizophrenia patients treated with clozapine(Yi et al., 2012). It is not clear whether PPAR γ agonists improve cognitive function in schizophrenia and disease and medicine differences should be considered at the same time. More works with larger sample size and longer treatment duration are needed.

### Others

#### SGLT2 Inhibitors

Sodium-glucose co-transporter (SGLT) inhibitors are one of the newly developed medications used to treat T2DM(Chao and Henry, 2010). SGLT 2 inhibitors, such as dapagliflozin and empagliflozin, decrease renal glucose reabsorption, resulting in enhanced urinary glucose excretion and subsequent reductions in plasma glucose and glycosylated hemoglobin concentrations(Nauck, 2014). SGLT2 inhibitors could improve insulin sensitivity and glucose homeostasis via reducing glucotoxicity, improving β cell function, reducing oxidative damages and inflammatory processes and inducing caloric disposition and weight loss(Yaribeygi et al., 2020). Dapagliflozin could improve brain insulin sensitivity, alleviate brain mitochondrial dysfunction, attenuate brain apoptosis and brain inflammation, and preserve hippocampal synaptic plasticity(Sa-nguanmoo et al., 2017). However, no sufficient preclinical and powered clinical studies have elucidated the cognitive benefit of SGLT2 inhibitors in AD and schizophrenia.

#### Quercetin

As a natural pigment, quercetin is a flavonoid with abundant biological activities such as antiviral, anticancer, antioxidation, anti-inflammatory, and antidiabetic activity(Batiha et al., 2020). The antidiabetic effects of quercetin involve the inhibition of intestinal glucose absorption, insulin secretory and insulin-sensitizing activities as well as the enhancement of glucose utilization in peripheral tissues(Eid and Haddad, 2017). A meta-analysis supported that the flavonoid quercetin lowered the serum glucose level at dose of 10, 25 and 50 mg/kg in diabetic animals(Bule et al., 2019). Additionally, quercetin could suppress endoplasmic reticulum stress and tau phosphorylation through its AMPK activity to provide potential cognitive benefit(Chen et al., 2016). Quercetin exhibited neuroprotection through its effects on sodium channels of neurons(Yao et al., 2010), and modulating Akt signaling pathway to inhibit neuronal apoptosis(Pei et al., 2016). Quercetin ameliorated cognitive and emotional impairment, reversed brain levels of β-amyloidosis and tauopathy, attenuated mitochondrial dysfunction and alleviated astrogliosis and microgliosis in AD mice model(Wang et al., 2014; Sabogal-Guáqueta et al., 2015). Quercetin exhibited spatial learning and memory improvement in Parkinson’s Disease model via its antioxidant effect resulting in the promotion of neuron survival(Sriraksa et al., 2012). Quercetin also improved the chronic unpredicted stress-mediated memory dysfunction through normalizing metabolic aberration, attenuating insulin resistance, elevating hippocampal GLUT4 levels, and maintaining neuronal integrity(Mehta et al., 2017). In a ketamine model of schizophrenia, quercetin showed the potential to improve cognitive deficits, partly owing to its ability to scavenge free radicals and its high antioxidant capacity(Mert et al., 2019). However, a clinical study failed to demonstrate the effects of a 12-week quercetin supplementation program on cognitive function(Broman-Fulks et al., 2012). There may exist some gaps when translate in vitro studies and in vivo experiments into clinical situation and more clinical studies are needed to determine whether quercetin shows cognitive benefits.

#### Sulforaphane

Sulforaphane is a compound with multiple bioactivities such as antidiabetic and antioxidant effects. Sulforaphane could improve glucose tolerance through up-regulation of insulin signaling involving IRS-1/Akt/GLUT 4 pathway in muscle(Xu et al., 2018). Sulforaphane also attenuated glucose intolerance and affected GLUT3 expression in the cerebral cortex and hypothalamus(Souza et al., 2013). Sulforaphane mitigated cognitive decline which was associated with an upregulation of the nuclear accumulation and transcriptional function of the nuclear factor erythroid 2-related factors (Nrf2) and an enhancement of the antioxidative response in the hippocampus(Pu et al., 2018). The transcription factor Nrf2 functions at the interface of cellular redox and intermediary metabolism(Dinkova-Kostova et al., 2018), which modulates the expression of defensive genes encoding detoxifying enzymes and antioxidant proteins(Kaspar et al., 2009). In addition to regulating the expression of antioxidant genes, Nrf2 has also been shown to exhibit anti-inflammation effects and modulate mitochondrial function and biogenesis(Brandes and Gray, 2020). Sulforaphane prevented olanzapine-induced glucose and lipid metabolism dysregulation in preclinical studies(Isaacson et al., 2020). In phencyclidine administration mice, sulforaphane ameliorated hyperlocomotion and pre-pulse inhibition deficits(Shirai et al., 2012). Additionally, sulforaphane exhibited prophylactic and therapeutic effects in phencyclidine-induced cognitive deficits, which was substantiated by the results of Novel Object Recognition test(Shirai et al., 2015). A small open label clinical research suggested that sulforaphane had the potential to improve some domains of cognitive function in schizophrenia, although it failed to detect any statistical differences of the serum level of BDNF after taking sulforaphane for 8 weeks to baseline(Shiina et al., 2015). A 6-month clinical trial was conducted to evaluate whether adding sulforaphane treatment would benefit the negative symptoms and cognitive function in individuals with schizophrenia (clinicaltrials.gov: NCT04521868). Larger randomized, double-blind, placebo-controlled trials conducted over longer periods are needed to determine the efficacy and safety of sulforaphane.

## Conclusion

Cognitive deficits occur in neurodegenerative and neuropsychiatric diseases including AD and schizophrenia. Insulin resistance is a shared metabolic comorbidity of these two chronic diseases. The disruption of insulin action in the brain is considered as a potentially important pathophysiological mechanism of cognitive impairment via deficits in neuronal structure and function, impaired synaptic plasticity, brain mitochondrial dysfunction, increased oxidative stress and inflammation. Intrinsic abnormal glucose metabolism would predispose AD and schizophrenia patients to cognitive deficits and current therapeutic measures cannot achieve expected therapeutic efficacy. Therapeutic interventions may be effective if both the periphery and brain glucose metabolic disruptions are alleviated for effective results. Antidiabetic agents have gained public and scientific attention for its potential as a cognitive enhancement drug and is currently being examined in in vitro experiment, in preclinical animal studies and in clinical trials around the word. The molecular mechanism and preclinical animal models suggest that the repositioning of antidiabetic drugs is a promising opportunity to alleviate cognitive deficits in AD and schizophrenia. However, there may exist some gaps when translate experimental results into application. Drugs specifically designed to target brain insulin resistance may exert beneficial effects on cognition. Furthermore, the pathogenesis and clinical heterogeneity of cognitive impairment remain complex. A better understanding of the relationship between brain insulin resistance and cognitive impairment will help us to design future research. More works are urgent to explore the nature and corelates of brain insulin resistance and cognitive deficits in schizophrenia. For example, preclinical models are expected to discover the molecular and cellular mechanisms of cognitive deficits in schizophrenia, such as cerebral organoids derived from human embryonic stem cells and patient-derived induced pluripotent stem cells from schizophrenia, which are able to clarify the pathogenesis of cognitive deficits and the role of brain insulin resistance to exclude the effects of antipsychotics on this relationship. Additionally, radiotracer or detection methods are expected to clarify the central insulin action in patients with schizophrenia. Some anti-diabetic agents may fail as cognitive enhancement strategies for a number of reasons, such as poor penetrance of the blood–brain barrier and ineffectiveness in reducing brain insulin resistance in vivo. Therefore, works are needed to examine the effect of drugs that are able to increase the sensitivity of insulin receptors in the brain. Besides, multi-site large-scale randomized controlled trails are needed to examine the effects of antidiabetic medications on cognition in schizophrenia. In the future, more works are expected to elucidate the relationship between brain insulin resistance and cognitive dysfunction in schizophrenia leading to the development of novel interventions to alleviate cognitive deficits in schizophrenia to benefit patients.
